# Insight into the Selectivity of the G7-18NATE Inhibitor Peptide for the Grb7-SH2 Domain Target

**DOI:** 10.3389/fmolb.2017.00064

**Published:** 2017-09-26

**Authors:** Gabrielle M. Watson, William A. H. Lucas, Menachem J. Gunzburg, Jacqueline A. Wilce

**Affiliations:** Biomedicine Discovery Institute, Department of Biochemistry and Molecular Biology, Monash University, Clayton, VIC, Australia

**Keywords:** Grb7, SH2 domain, inhibitor specificity, BC loop, peptide inhibitor

## Abstract

Growth factor receptor bound protein 7 (Grb7) is an adaptor protein with established roles in the progression of both breast and pancreatic cancers. Through its C-terminal SH2 domain, Grb7 binds to phosphorylated tyrosine kinases to promote proliferative and migratory signaling. Here, we investigated the molecular basis for the specificity of a Grb7 SH2-domain targeted peptide inhibitor. We identified that arginine 462 in the BC loop is unique to Grb7 compared to Grb2, another SH2 domain bearing protein that shares the same consensus binding motif as Grb7. Using surface plasmon resonance we demonstrated that Grb7-SH2 binding to G7-18NATE is reduced 3.3-fold when the arginine is mutated to the corresponding Grb2 amino acid. The reverse mutation in Grb2-SH2 (serine to arginine), however, was insufficient to restore binding of G7-18NATE to Grb2-SH2. Further, using a microarray, we confirmed that G7-18NATE is specific for Grb7 over a panel of 79 SH2 domains, and identified that leucine at the βD6 position may also be a requirement for Grb7-SH2 binding. This study provides insight into the specificity defining features of Grb7 for the inhibitor molecule G7-18NATE, that will assist in the development of improved Grb7 targeted inhibitors.

## Introduction

For over 20 years, Src homology 2 (SH2) domains have been explored as targets for the development of potential therapeutics (Kraskouskaya et al., 2013; Morlacchi et al., 2014). SH2 domains mediate the formation of protein complexes by recognizing phosphorylated tyrosines (pY) on target tyrosine kinases and subsequently mediating their downstream effects. They are utilized by proteins with a range of functions, including enzymes such as the PLCγ and the Src kinase (Rebecchi and Pentyala, 2000; Roskoski, 2004), transcriptional regulators including STAT 1-6 (Darnell, 1997) and adaptor proteins including growth factor receptor bound protein 7 (Grb7) (Han et al., 2001). The reversible association of the SH2 domain/pY complex allows for the efficient regulation of signaling and is therefore frequently found in proteins mediating intracellular signaling pathways—including those regulating proliferation, migration and growth (Schlessinger and Lemmon, 2003). It is these same signaling networks that are susceptible to dysregulation, leading to the amplification and transmission of signals that drive cells into a cancerous state. Thus, targeting SH2 domains can serve as an effective point for the development of anti-cancer agents that effectively block the downstream effects of signaling.

Grb7 has been identified as a therapeutic target due to its role in the progression of a number of cancers including breast, pancreatic, ovarian and oesophageal cancers (Stein et al., 1994; Tanaka et al., 1997, 2006; Wang et al., 2010). In HER2+ breast cancer, Grb7 is co-overexpressed with HER2, leading to enhanced tumorigenesis and cell proliferation (Bai and Luoh, 2007; Chu et al., 2010; Pradip et al., 2013). While HER2 has a well-known role in breast cancer progression, overexpression of Grb7 has now also been identified as a significant predictor for reduced cancer-free periods and a worse prognosis for breast cancer patients (Ramsey et al., 2011). In pancreatic, oesophageal and triple negative breast cancers, Grb7 drives migratory and invasive events, with Grb7 knockdown inhibiting these processes when tested *in vitro* (Tanaka et al., 1997, 2000, 2006; Giricz et al., 2012). Furthermore, a significant relationship has been identified between Grb7 expression and tumor metastasis in pancreatic and esophageal cancers (Tanaka et al., 1997, 2006).

Grb7 has a multi-domain architecture consisting of an N-terminal proline rich domain, a Ras-associating (RA) domain, pleckstrin homology (PH) domain, a between the PH and SH2 (BPS) domain, and lastly a C-terminal SH2 domain (Shen and Guan, 2004). It is via the SH2 domain that Grb7 interacts with phosphorylated tyrosine kinases including growth factor receptors such as HER2, HER3 and EGFR, as well as cytoplasmic kinases including the focal adhesion kinase (FAK) (Stein et al., 1994; Daly et al., 1996; Han and Guan, 1999). Through these interactions, Grb7 mediates signaling networks controlling proliferation, migration and growth, making the Grb7-SH2 domain an attractive candidate for the development of targeted inhibitors (Han and Guan, 1999; Pero et al., 2003; Pradip et al., 2013).

SH2 domains are extremely prevalent in the proteome, with over 110 proteins bearing the domain. Thus, ensuring target selectivity is a critical aspect of the development of molecules targeting SH2 domains. Despite the high number, SH2 domains display exquisite selectivity for their substrates (Pawson, 2004). SH2 domains contain a well-characterized positively charged cleft for the pY to bind, but it is typically the residues C-terminal to the target pY that dictate the binding specificity for a substrate (Songyang et al., 1993). It has been determined that individual SH2 domains recognize characteristic binding motifs. In the case of Grb7 this motif has been identified as pYXN, where any residue is accommodated at the +1 position, and an asparagine is preferred at the +2 position (Pero et al., 2003). The SH2 domains of Grb2 and Grb7 have low amino acid identity (29%), however, the Grb2-SH2 also recognizes this binding motif, and both adaptor proteins bind to HER2 at pY1139 (Janes et al., 1997). Despite this binding similarity, Grb7 and Grb2 can have remarkable selectivity and an inhibitor has been developed that specifically binds and inhibits Grb7-SH2 (Pero et al., 2002).

The cyclic, non-phosphorylated peptide (named G7-18NATE (cyclo-(CH_2_CO-WFEGYDNTFPC)-amide) was developed via phage display and was found to specifically decrease binding between Grb7 and tyrosine phosphorylated HER family members in breast cancer cell extracts, but have no effect on the interaction between Grb2 and HER3 (Pero et al., 2002). When G7-18NATE was attached to the Penetratin cell permeability sequence, the peptide inhibited growth, migration and proliferation in breast cancer cell lines, and displayed synergistic effects on cell proliferation with the currently available chemotherapeutics Doxorubicin and Trastuzumab (Pero et al., 2007; Pradip et al., 2013). G7-18NATE-Penetratin was also shown to specifically inhibit the interaction between Grb7 and FAK, but not interfere with the Grb2/FAK or Grb2/EGFR interactions (Tanaka et al., 2006). This specificity for the Grb7-SH2 has also been demonstrated *in vitro* with surface plasmon resonance (SPR) experiments confirming that G7-18NATE specifically binds to Grb7-SH2 preferentially over the Grb2-SH2 domain (Gunzburg et al., 2012). G7-18NATE also displayed minimal binding to the SH2 domains of Grb10 and Grb14, proteins that share the same domain structure as Grb7 (with the three Grbs collectively termed the Grb7 family). Derivatives of G7-18NATE have also been developed that bind to the Grb7-SH2 domain with higher affinity than G7-18NATE (*K*_D_ = 0.83 μM) but with the equivalent specificity (Watson et al., 2015; Gunzburg et al., 2016).

**Figure 1:**
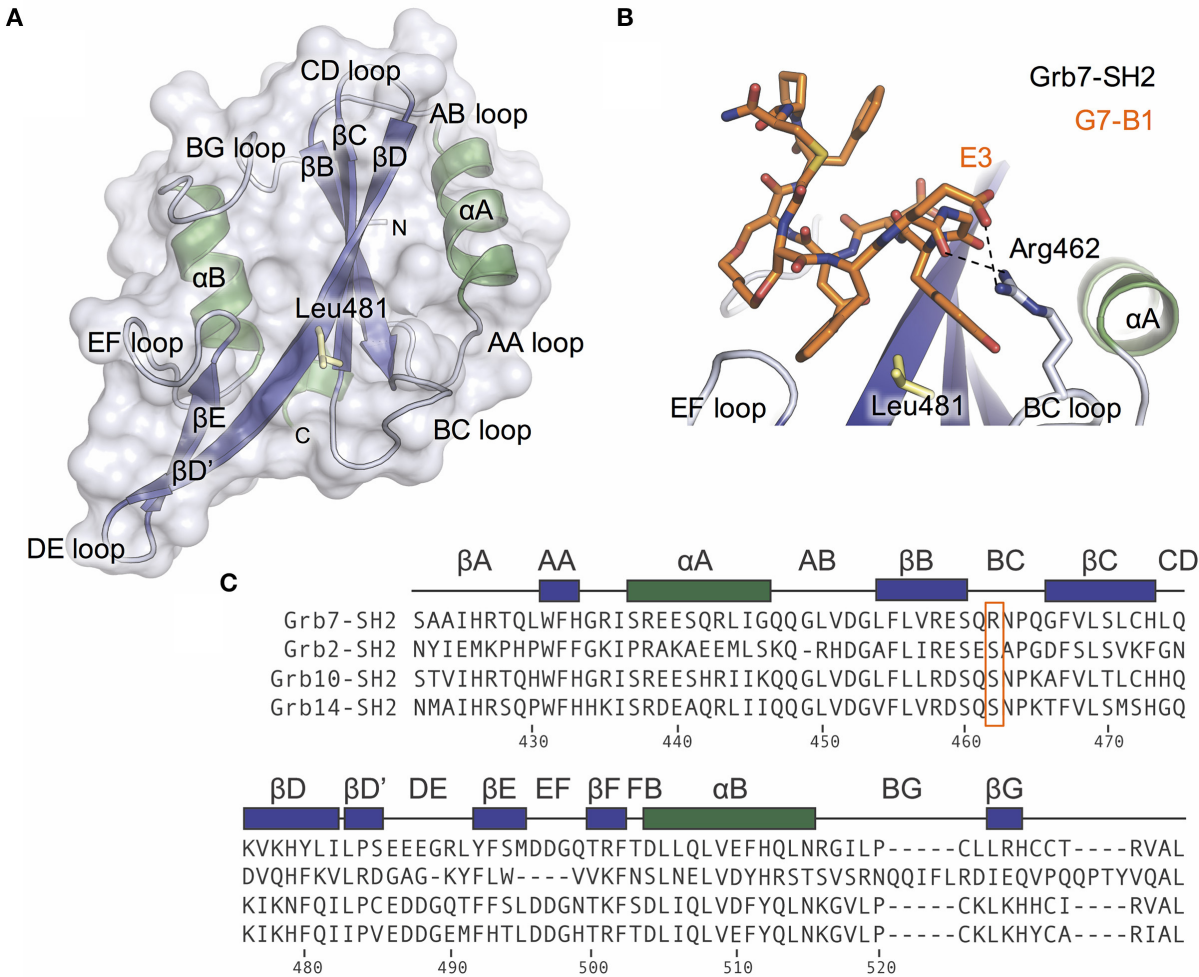
The Grb7 specific amino acid Arg462 may contribute to the Grb7-SH2/G7-18NATE interaction. **(A)** Overview of the Grb7-SH2 domain with SH2 domain nomenclature displayed. Surface representation and loops displayed in gray, α–helices in green and β-sheet in purple. The previously described specificity defining Leu481 is displayed as yellow sticks; **(B)** Close-up of the interaction of the G7-B1 bicyclic peptide (orange sticks) with the Grb7-SH2 domain colored as in **(A)**. Hydrogen bonds between the Grb7-SH2 Arg462 and the peptide E3 are displayed as dashed lines (PDB ID: 5EEQ); **(C)** Protein sequence alignment of the SH2 domains of Grb2, Grb7, Grb10 and Grb14, generated using Clustal Omega (Sievers et al., 2011). Secondary structural elements for the Grb7-SH2 domain are displayed above the alignment at the corresponding position, and sequence numbering for the Grb7-SH2 displayed below the alignment. Grb7 amino acids that form hydrogen bond interactions with the G7-18NATE peptide are identified with filled circles and additional amino acids that contribute to the buried surface area are identified with empty circles. The unique Grb7-SH2 arginine at position 462 (BC2) is highlighted by an orange box.

While it has been demonstrated repeatedly that G7-18NATE and its derivatives are specific for Grb7, there has been little investigation into the molecular basis of Grb7 ligand selectivity, except for analysis of the βD6 amino acid (Leu481 in Grb7). It is important to note that the nomenclature to be used to describe SH2 domain secondary structure is as derived by Eck et al. as illustrated in Figure 1A (Eck et al., 1993). While Grb10 and Grb14 possess a polar glutamine at the βD6 position, Grb7 has a hydrophobic leucine residue. When Leu481 in Grb7-SH2 was mutated to glutamine, binding to the HER2 receptor was completely abolished (Janes et al., 1997). Conversely, the opposite mutation in Grb14 (glutamine to leucine) imparted binding of Grb14 to the HER2 receptor. Together this established the importance of a leucine at the βD6 position for Grb7-SH2 domain interactions with its target motif. In the case of the Grb2-SH2 domain, however, the βD6 amino acid is a lysine and this would be predicted to interfere with ligand binding. Yet some binding to Grb2-SH2 domain by G7-18NATE still takes place, showing that it is not only the βD6 amino acid that dictates G7-18NATE binding (Gunzburg et al., 2012).

The structure of the Grb7-SH2 domain in complex with G7-18NATE revealed the amino acid residues forming the binding site for the peptide ligand (Ambaye et al., 2011b). It showed the way in which G7-18NATE Y5 and F2 (note that single letter code will be used in reference to the peptide ligand amino acids for clarity) straddle the Grb7-SH2 domain Leu481 residue, helping to visualize the role of this amino acid in the specificity of the interaction. Leu481, and other Grb7-SH2 domain amino acids that form hydrogen bond interactions or contribute to the buried surface area with G7-18NATE are indicated in Figure 1C. For the most part, these residues are conserved between Grb7-SH2 domain and the closely related Grb10, Grb14, and Grb2 SH2 domains and, thus, do not help to explain the basis for Grb7 ligand specificity. Only the βE4 methionine and EF4 glutamine are unique to the Grb7-SH2 domain and may act alongside Leu481 to contribute to ligand specificity.

In this study, we identified an additional amino acid in the Grb7-SH2 BC loop that is involved in binding to G7-18NATE and potentially also contributes to Grb7-SH2 domain binding specificity. Based on another Grb7-SH2/peptide complex X-ray crystal structure, we postulated that the Grb7 BC2 arginine contributes to the Grb7-SH2/peptide interaction. In Grb2, Grb10, and Grb14 the BC2 residue is a serine. Using mutational analysis we demonstrate that this Grb7 residue does indeed contribute to the G7-18NATE interaction, and thus may dictate some of the Grb7 selectivity. We identified that mutating the BC2 residue in the Grb2-SH2 from serine to arginine is not sufficient to confer binding to G7-18NATE indicating that other Grb7 amino acids also contribute to binding strength and specificity. Lastly, using a pY micro-array we confirmed that G7-18NATE is specific for Grb7 compared to a panel of 79 SH2 domains, providing extra verification of the specificity of this peptide. Together this study provides insight into the specificity of G7-18NATE for Grb7 which is critical for the development of effective therapeutics targeted to the Grb7-SH2 domain signaling in cancer.

## Materials and methods

### Protein and peptide preparation

Grb7-SH2 and Grb2-SH2 were prepared as GST fusion proteins and expressed and purified as previously described (Watson et al., 2015). Briefly, Grb7-SH2 (residues 415-532) and Grb2-SH2 (residues 58-160) were incorporated into the pGex2T plasmid and expressed in BL21(DE3)pLysS competent cells following IPTG induction (0.4 mM) at 25°C. Subsequently, glutathione affinity chromatography and size exclusion chromatography were used to purify the soluble GST tagged proteins. To generate the Grb7-SH2 R462S and Grb2-SH2 S90R mutants, site-directed mutagenesis was utilized (Liu and Naismith, 2008). For this, the wild-type SH2 domain constructs were used as templates for polymerase chain reaction with the primers listed in Supplementary Material. The parent plasmid was subsequently removed by treatment with *Dpn1* and the presence of the mutation verified by DNA sequencing. The mutant proteins were expressed and purified as per the wild-type proteins. GST alone was purified similarly to the GST-SH2 domain proteins with the exception that size exclusion chromatography was not necessary for purification.

G7-18NATE (cyclo-(CH_2_CO-WFEGYDNTFPC)-amide) was synthesized using standard Fmoc-chemistry and purchased from Purar Chemicals (Australia). The synthesis of G7-18NATE-PB ((cyclo-(CH_2_CO-WFEGYDNTFPC-RRMKWKKK(Biotin))-amide)) has been previously described (Ambaye et al., 2011a). The purity of both peptides was >95% as determined using LC-MS.

The final solution concentration of all proteins and peptides used in this study were determined spectroscopically at 280 nm using extinction coefficients predicted by the ProtParam server (Gasteiger et al., 2005).

### Binding studies using surface plasmon resonance

SPR experiments were performed on a Biacore T100 using CM5 series S sensor chips. The GST tagged proteins were immobilized onto the sensor chip surface by amine coupling an anti-GST antibody to the surface of the chip. For this, firstly the chip was activated by 0.4 M 1-ethyl-3-(3-dimethylaminopropyl)carbodiimide hydrochloride and 0.1 M N-hydroxysuccinimide. The polycloncal antibody (Abcam) was passed over the chip surface at 30 μg.mL^−1^ before the flow cells were blocked with 1 M ethanolamine. The running buffer used for the immobilization contained 50–150 mM NaPO_4_, 150 mM NaCl and 1 mM DTT (pH 7.4), with levels reaching between 3289 RU and 6324 RU. The GST fusion proteins were passed over the active chip surface at 0.7–0.9 μM to achieve final immobilization levels of 838-1283 RU. GST alone was immobilized on the reference flow cell (462-616 RU) as a negative control to allow for double referencing. Lyophilized G7-18NATE was resuspended in running buffer containing 150 mM NaPO_4_, 150 mM NaCl and 1 mM DTT (pH 7.4) and injected over the chip surface for 60 s at 30 μL.min^−1^ in triplicates. Due to machine error some injections were removed before analysis due to presence of spikes that rendered the sensorgrams unable to be interpreted. The interpretable data were analyzed using Scrubber2.0 (BioLogic Softward, Campbell, ACT, Australia) and GraphPad Prism.

### SH2 domain pY microarray

The microarray was conducted by the Protein Array and Analysis Core at the MD Anderson Cancer Center (University of Texas, USA) using standard protocols. Briefly, lyophilized G7-18NATE-PB (100 μg) was resuspended in PBST and incubated with the nitrocellulose slide pre-spotted with GST-fusion SH2 domains (full list provided as Supplementary Material). Each SH2 domain was spotted in duplicate.

## Results

### The unique Grb7-SH2 BC2 amino acid engages E3 in the G7-B1 peptide

We recently characterized the interaction between the Grb7-SH2 and a G7-18NATE derivative peptide, G7-B1 (B for bicyclic) (Gunzburg et al., 2016). In the course of our investigation, we solved the X-ray crystal structure of the Grb7-SH2/G7-B1 complex to 1.6 Å (PDB ID: 5EEQ). This revealed an interesting feature potentially underlying binding specificity of the Grb7 targeting peptides. We identified that an arginine at the BC2 position (Arg462) in the Grb7 BC loop reaches up and hydrogen bonds with the G7-B1 E3 backbone carbonyl and forms a salt bridge with the E3 sidechain carboxylate (Figure 1B; Gunzburg et al., 2016).

A sequence alignment of the SH2 domains of Grb2, Grb7, Grb10, and Grb14 revealed that an arginine at this BC2 position is unique to Grb7 with Grb10, Grb14, and Grb2 possessing a serine at the equivalent position (Figure 1C). Although the Arg462/E3 interaction has not been clearly observed in other Grb7-SH2/peptide crystal structures (PDB ID:3PQZ Ambaye et al., 2011b; PDB ID 4X6S Watson et al., 2015; Gunzburg et al., 2016), we postulated that this interaction might occur transiently and help to define the molecular basis of binding specificity of G7-18NATE for Grb7.

### Arg462 contributes to the binding affinity between Grb7-SH2/G7-18NATE

To test this hypothesis, we mutated the Grb7-SH2 R462 (BC2) amino acid to a serine (the corresponding residue in the Grb2, Grb10. and Grb14 SH2 domains). Using SPR, we directly compared the binding of the cyclic peptide G7-18NATE to the Grb7-SH2 wild-type (WT) and the mutated Grb7-SH2 (R462S). The Grb7-SH2 proteins were prepared as GST fusions to enable efficient immobilization on the sensor chip surface via anti-GST antibodies. The G7-18NATE peptide was flowed over the sensor chip surface and the measured response at equilibrium used to construct binding curves that were fitted by a single-site binding model.

**Figure 2:**
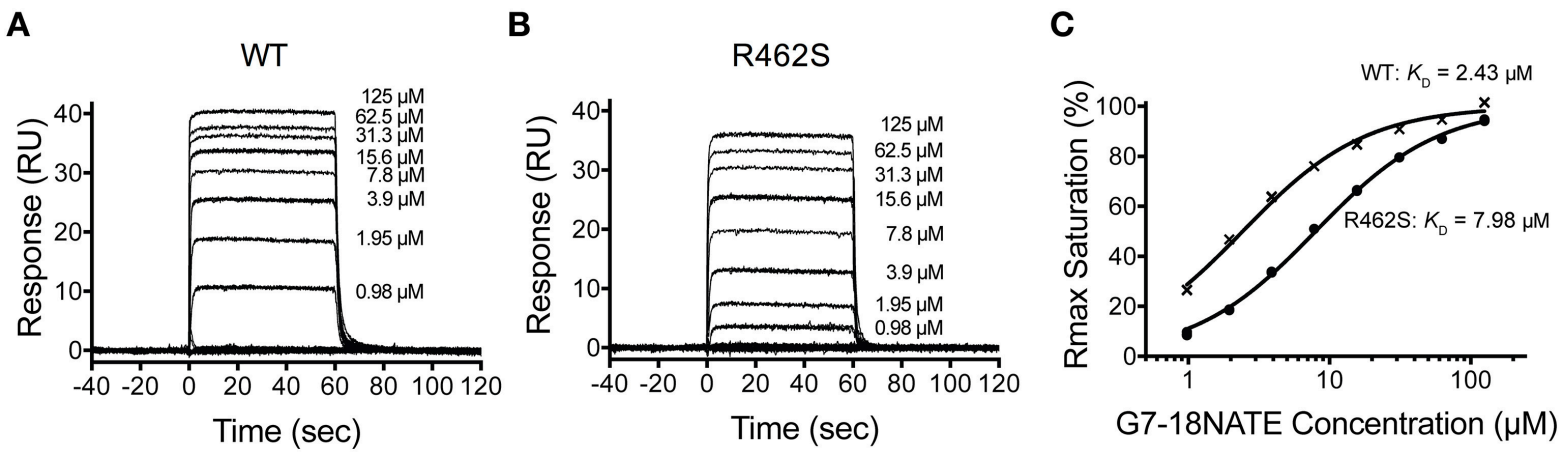
The Grb7 R462S mutant has decreased binding for G7-18NATE. Sensorgrams of the interaction between G7-18NATE with either wild-type (WT) Grb7-SH2 **(A)** or mutated (R462S) Grb7-SH2 **(B)** with the concentrations of peptide tested displayed on the right of the sensorgram; **(C)** The corresponding binding curves were calculated from the responses at equilibrium and fitted by a 1:1 interaction model. All data points from the repeated experiments are displayed in the binding profiles.

Upon injection of G7-18NATE, equilibrium was rapidly reached for both WT Grb7-SH2 (Figure 2A) and the mutated R462S Grb7-SH2 (Figure 2B), followed by rapid dissociation after the 60 s injection was complete. Excellent fits by a 1:1 interaction model were obtained with a *R*^2^ of 0.99 for both interactions. The binding curves indicated that the R462 of the BC loop did indeed contribute to the binding affinity of the G7-18NATE/Grb7-SH2 interaction. While G7-18NATE bound to WT Grb7-SH2 with a *K*_D_ of 2.43 μM, the R462S mutant had a 3.3-fold reduction in binding affinity for G7-18NATE, with a *K*_D_ of 7.97 μM (Figure 2C; Table 1). This suggested that the BC2 arginine does indeed engage with the peptide ligand, and therefore may impart some of the specificity of Grb7 for its substrates.

**Table 1:** SPR investigations into the specificity of G7-18NATE for the Grb7-SH2.

**Protein**	**Equilibrium Dissociation Constant (μM)^#^**	**Rmax (RU)**
Grb7-SH2 WT	2.43 ± 0.01	39.7 ± 0.05
Grb7-SH2 R462S	7.98 ± 0.03	37.9 ± 0.04
Grb2-SH2 WT	>125	14.2 ± 0.9
Grb2-SH2 S90R	>125	41.0 ± 3

# *Equilibrium dissociation constants were calculated from fits by a single-site binding model. Errors displayed are the standard error of the fits*.

### The corresponding Grb2 mutation is insufficient to confer binding to G7-18NATE

**Figure 3:**
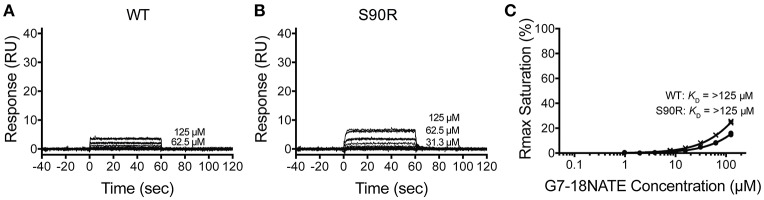
The Grb2 S90R mutant has minimal binding to G7-18NATE. Sensorgrams of the interaction between G7-18NATE with either wild-type (WT) Grb2-SH2 **(A)** or mutated (S90R) Grb2-SH2 **(B)** with the concentrations of peptide tested displayed on the right of the sensorgram (lower concentrations omitted for clarity); **(C)** The corresponding binding curves were calculated from the response at equilibrium and fitted by a 1:1 interaction model. All data points from the repeated experiments are displayed in the binding profiles.

We next postulated that the opposite mutation at the BC2 position in the Grb2-SH2 (serine to arginine) could confer binding of the Grb2-SH2 to G7-18NATE. This would indicate the degree to which Arg462 in Grb7 is responsible for binding specificity. We therefore generated the Grb2-SH2 S90R (BC2) mutant protein using site-directed mutagenesis and tested its binding to G7-18NATE using the same SPR experimental procedure described above. As shown in Figure 3A, there was only a low binding response to the Grb2-SH2 WT following injection of G7-18NATE over the chip surface, reflecting low binding affinity as previously established for Grb2-SH2 compared to Grb7-SH2 (Gunzburg et al., 2012). The binding to G7-18NATE by Grb2-SH2 S90R, however, also gave rise to low responses, though slightly higher than seen for Grb2-SH2 WT (Figure 3B). Excellent fits were obtained by a single-site binding model (*R*^2^ = 0.99), however the calculated equilibrium dissociation constant was higher than the highest peptide concentration measured (125 μM; Figure 3C). Therefore, we could not reliably determine differences in the *K*_D_ between WT Grb2-SH2 and the mutated Grb2-SH2. This demonstrated, nevertheless, that the incorporation of the arginine at the BC2 position was not sufficient to significantly increase the binding of Grb2-SH2 to G7-18NATE.

### G7-18NATE is specific for the Grb7-SH2 compared to a panel of SH2 domains

In the course of our analysis of G7-18NATE and its derivatives, specificity was assessed by comparing the binding of the peptides to the SH2 domains of Grb2, Grb7, and Grb10, and in some instances Grb14-SH2 (Watson et al., 2015; Gunzburg et al., 2016). While Grb2 shares the same consensus pYXN binding motif, Grb10 and Grb14 share significant sequence identity (approximately 70%) with Grb7-SH2 (Daly et al., 1996). We therefore considered that these were the most closely related SH2 domains for assessment of specificity for Grb7-SH2 using SPR experiments.

**Figure 4:**
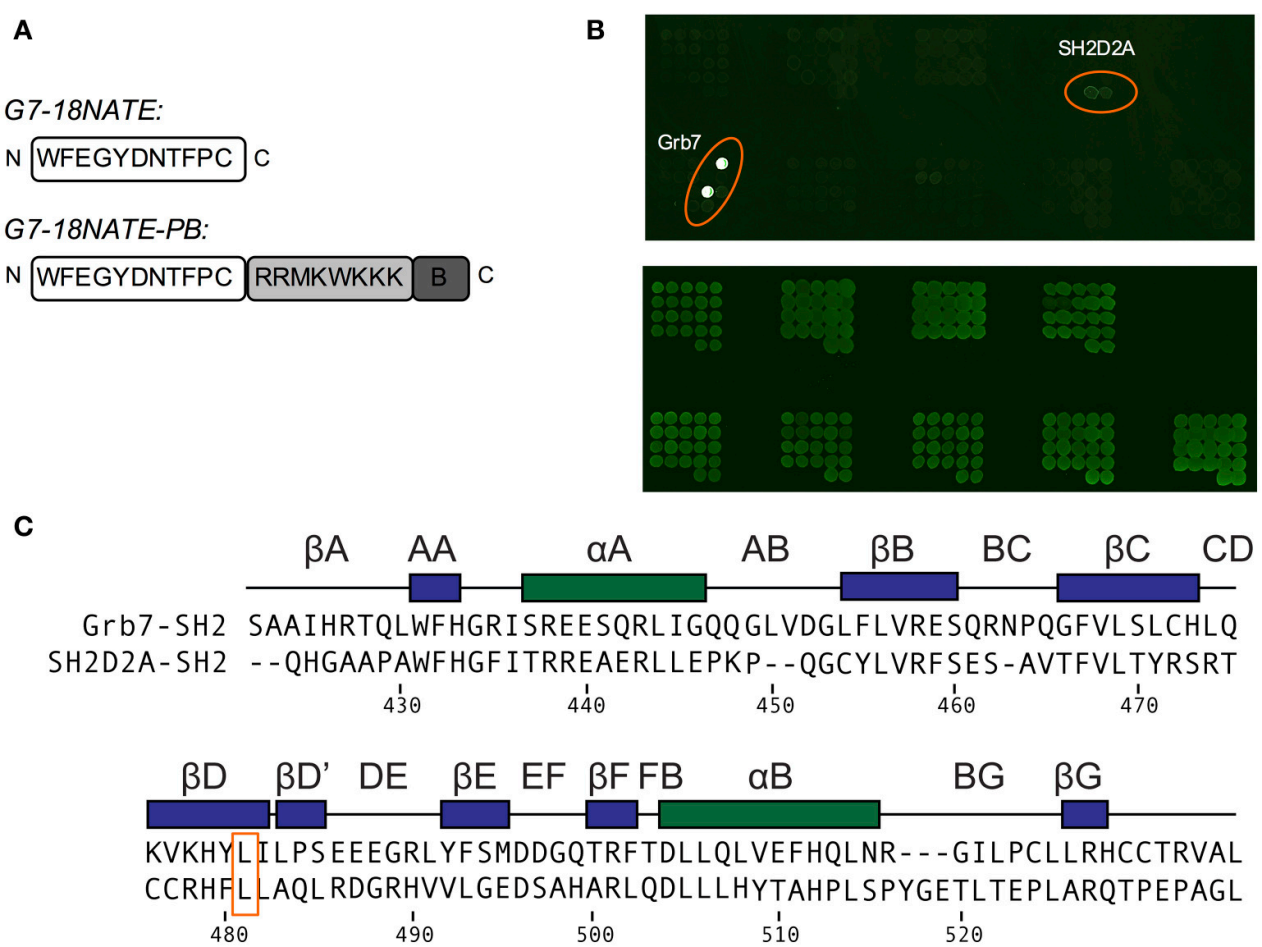
G7-18NATE-PB is specific for Grb7 over a panel of SH2 domains. **(A)** Schematic of G7-18NATE and G7-18NATE-PB with the single letter amino acid code and the amino (N) and carboxy (C) termini displayed. (B) Represents the biotin moiety. **(B)** SH2 domain protein microarray. Top Panel: following treatment with 100 μg of G7-18NATE-PB; Bottom Panel: Anti-GST antibody loading control to show equivalent amounts of SH2 domain and the positions of the microarray spots. A list of SH2 domains in the microarray is provided as Supplementary Information. **(C)** Protein sequence alignment of the SH2 domains of Grb7 and Sh2D2A, generated using Clustal Omega (Sievers et al., 2011). Secondary structural elements for the Grb7-SH2 domain are displayed above the alignment at the corresponding position, and sequence numbering for the Grb7-SH2 displayed below the alignment. The shared leucine at the βD6 position is highlighted by an orange box.

In order to determine whether there were other SH2 domains that G7-18NATE could bind to that we had not yet encountered, we utilized the pY reader protein microarray at the Protein Array and Analysis Core (MD Anderson Cancer Center). Here, a biotinylated G7-18NATE derivative (G7-18NATE-PB) was tested for binding against 79 SH2 domains (a complete list of tested SH2 domains is provided as Supplementary Material). The G7-18NATE derivative also contained a shortened Penetratin sequence (displayed as a schematic in Figure 4A), and the presence of this sequence has been previously demonstrated not to interfere with G7-18NATE binding to the Grb7-SH2 (Ambaye et al., 2011a; Watson et al., 2017). As shown in Figure 4B, it was clear that G7-18NATE-PB preferentially binds to the Grb7-SH2 over the suite of SH2 domains. Although this array was not quantitative, the level of fluorescence was found to be significantly higher for the positions containing the Grb7-SH2, indicative of peptide binding. This was not due to differences in the amount of protein blotted on the array, as similar fluorescence levels were detected when probed with an anti GST antibody (Figure 4B).

There was no observation of fluorescence at the positions corresponding to Grb2, Grb10, or Grb14, consistent with our previously published SPR experiments (Gunzburg et al., 2012). Fluorescence was observed, although at much lower intensity, at the positions corresponding to a previously untested SH2 domain—that of SH2D2A. A sequence alignment of the SH2 domains of Grb7 and SH2D2A revealed that SH2D2A also contains a serine at the BC2 position and, strikingly, a leucine at the βD6 position (Figure 4C). Although this is not the only SH2 domain in the array that posseses a leucine at the βD6 position, it is suggestive that leucine at this position is also determining feature for binding the G7-18NATE peptide and its derivatives. Thus, as well as further confirming the specificity of the ligand G7-18NATE for the Grb7-SH2 domain, this experiment has also provided further insights into the defining features of Grb7 that affect ligand binding selectivity.

## Discussion

SH2 domains are frequently found in signaling pathways regulating critical processes that are perturbed in aggressive diseases like cancer. Due to this, and their well-characterized binding mode, a number of SH2 domains have been targeted for the development of novel therapeutics (Kraskouskaya et al., 2013; Morlacchi et al., 2014). However, with over 110 SH2 domains in the proteome, ensuring selectivity for the target SH2 domain is a significant challenge in the development of specific and potent inhibitors. Grb7 is a signaling adaptor protein that has been specifically targeted through the development of cyclic peptide inhibitors that are able to inhibit proliferation and migration in breast cancer cell lines and reduce tumor metastasis in a mouse model of pancreatic cancer (Tanaka et al., 2006; Pradip et al., 2013). The cyclic peptide, G7-18NATE, has been demonstrated to be specific for the Grb7-SH2 domain over the closely related SH2 domains of Grb2, Grb10, and Grb14, and this specificity has, to date, been attributed to the Grb7 specific leucine at the βD6 position (Janes et al., 1997; Gunzburg et al., 2012). Here, we have investigated whether additional amino acids also contribute to the specificity of Grb7 for its ligands.

Based on the high-resolution X-ray crystal structure of the Grb7-SH2/G7-B1 complex, we identified an arginine in the Grb7-SH2 BC loop that mediates additional hydrogen bond interactions with the inhibitor peptide. We identified that this arginine is unique to Grb7 compared to the SH2 domains of Grb2, Grb10 and Grb14 (where there is a serine at the comparable position) and considered that this interaction could contribute to the specificity for Grb7 of all the G7-18NATE derived peptide ligands. Using mutagenesis we henceforth determined that Arg462 contributes to the binding affinity between Grb7-SH2 and G7-18NATE. The converse mutation in the Grb2-SH2 domain did not confer binding to G7-18NATE, suggesting other amino acids also define Grb7 specificity. We also probed a panel of 79 SH2 domains with a biotinylated version of G7-18NATE and identified that the peptide is highly specific for the Grb7-SH2 domain. The only other SH2 domain that showed detectable binding by G7-18NATE was that of SH2D2A. Interestingly, while SH2D2A does not possess an arginine at the BC2 position it has a leucine at the βD6 position.

The initial observation that Arg462 may contribute to the binding interaction came from the Grb7-SH2/G7-B1 X-ray crystal structure (PDB ID: 5EEQ). In this structure, the Arg462 side-chain extends and engages the E3 backbone carbonyl in G7-18NATE, as well as the E3 sidechain carboxylic acid. The former of these interactions is likely to be more influential to binding. In previously described crystal structures of cyclic peptides bound to the Grb7-SH2 domain, the E3 sidechain is positioned in various rotamer conformations whereas Arg462 is frequently observed in the proximity of the E3 backbone carbonyl (Gunzburg et al., 2016). Furthermore, peptide ligands without glutamic acid at the E3 position have also been shown to bind the Grb7-SH2 domain (Ambaye et al., 2011b). This was initially identified using a constrained phage display library, enriched for a CX_1_FX_2_GYDNX_3_X_4_X_5_ motif, whereby tryptophan was selected in approximately 50% of clones at the corresponding E3 position. Thus, G7-18NATE derivatives that are modified at the E3 position are predicted to maintain the hydrogen bond interaction with the Grb7 Arg462 (BC2). It should be noted that the formation of this single hydrogen bond, that may only occur transiently (since it is not observed in all crystal structures), is consistent with the relatively small increase in affinity that is conferred. A 3.3-fold change in *K*_D_ corresponds to approximately only 1 kcal/mol change in ΔG, which is less than expected for a well formed hydrogen bond.

The G7-18NATE peptide and its derivatives are constrained in a β-turn conformation with extensive intra-molecular hydrogen bonds. A hydrogen bond between the F2 backbone carbonyl and the Y5 backbone amine positions the E3 backbone carbonyl for engagement with the Arg462 (BC2) sidechain. This suggests that the Arg462/substrate interaction may only occur in ligands that are naturally restricted in a β-turn conformation. To date, no X-ray crystal structures have been solved of Grb7 in complex with an *in vivo* binding partner (such as FAK or HER2) to determine whether or not Arg462 (BC2) contributes to the interaction with these molecules, and defines Grb7 selectivity for natural substrates.

We have also described how the introduction of the arginine mutation to the Grb2-SH2 at the BC2 position was insufficient to confer binding by the G7-18NATE peptide (although it may have made a contribution to binding to Grb10 or Grb14 that are more closely related to Grb7). This suggests that additional amino acids underlie the specificity of G7-18NATE for Grb7-SH2 over Grb2-SH2. This is supported by our microarray results where the only SH2 domain other than Grb7 to show detectable binding to G7-18NATE was that of SH2D2A, an SH2 domain that contains a leucine at the βD6 position. This finding is consistent with previous reports that report the contribution of leucine at the βD6 position to the specificity of Grb7. When Leu481 is mutated to glutamine (the corresponding amino acid in Grb14), binding to the HER2 receptor is lost, and conversely in Grb14, the glutamine to leucine mutation enables HER2 binding (Janes et al., 1997). The X-ray crystal structure of the Grb7-SH2/G7-18NATE complex revealed how a leucine at this position enhances the interaction (Ambaye et al., 2011b). Leucine at the βD6 position is not, however, sufficient for G7-18NATE binding to SH2 domains, as other SH2 domains in the microarray with leucine present at this position showed no ability to be bound by G7-18NATE. Clearly other amino acid residue differences contribute to binding specificity such as the βE4 methionine or EF4 glutamine that are unique to the Grb7-SH2 domain.

Thus, in this study we have demonstrated that Grb7 Arg462 contributes to G7-18NATE binding, but that specificity is mediated by multiple factors including the leucine at the βD6 position. We have also established that the G7-18NATE peptide is exquisitely specific for Grb7-SH2 over other SH2 domains. Together this provides insight for the development of even more potent ligands that target the Grb7-SH2 domain, and demonstrates that SH2 domains, despite their common structural features, are readily able to differentiate between ligands and thus have high potential as targets for therapeutics development.

## Author contributions

GW was responsible for the detailed experimental design, protein overexpression and SPR experiments, figure and manuscript preparation. WL undertook the cloning of SH2 domain mutants and was also involved in protein expression, purification and SPR experiments. MG provided SPR expertise and training. JW designed the conceptual framework for the experiments, oversaw the interpretation of the results and finalized the manuscript with the other authors.

### Conflict of interest statement

The authors declare that the research was conducted in the absence of any commercial or financial relationships that could be construed as a potential conflict of interest.

## Abbreviations

BPS: between PH and SH2 domain
FAK: focal adhesion kinase
Grb: growth factor receptor bound protein
PH: pleckstrin homology
pY: phosphorylated tyrosine
RA: ras-associating
SPR: surface plasmon resonance
SH2: src homology 2
WT: wild-type.
