# Supplementary material for: Insight into the Selectivity of the G7-18NATE Inhibitor Peptide for the Grb7-SH2 Domain Target
Source: Front Mol Biosci. 2017 Sep 26;4:64. doi: 10.3389/fmolb.2017.00064 (PMC5623053; doi:10.3389/fmolb.2017.00064)
Supplement: Supplementary file 1 [file Table1.pdf]

SUPPLEMENTARY INFORMATION

Insight into the selectivity of the G7-18NATE inhibitor peptide for the Grb7-SH2 domain target.

Gabrielle M. Watson, William A.H. Lucas, Menachem J. Gunzburg and Jacqueline A. Wilce.

A. List of Primers

Primer Name	Sequence (5' – 3')
Grb7 R462S Forward	AG AGT CAG **TCT** AAC CCC CAG GGC TTT GTC CTC TCT TTG TGC CAC CTG CAG
Grb7 R462S Reverse	TG GGG GTT **CGC** CTG ACT CTC CCG GAC CAG GAA CAG GCC
Grb2 S90R Forward	AG AGT GAG **CGC** GCT CCT GGG GAC TTC TCC CTC TCT GTC AAG TTT GG
Grb2 S90R Reverse	CC AGG AGC **GCG** CTC ACT CTC TCG GAT AAG AAA GGC CCC ATC GT

B. Table of SH2 domain GST fusion proteins tested in the pY micro-array

Phospho-Tyrosine Binding SH2 Domain Array 2.0

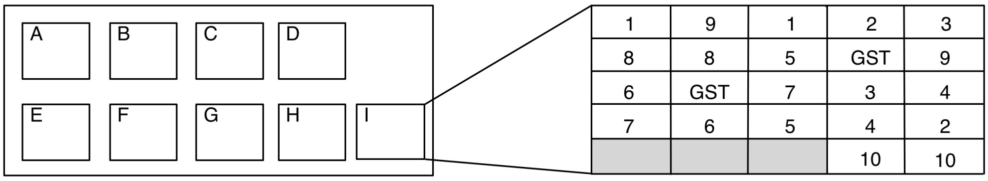

<u>Kinases</u><br>A1 LYN (P07948)<br>A2 HCK (P08631)<br>A3 LCK (P06239)<br>A4 BLKSH2 (P51451)<br>A5 FYN (P06241)<br>A6 YES (P07947)<br>A7 SRC (P12931)<br>A8 BRKPTK6 (Q13882)<br>A9 FRK (P42685)<br>A10 SRMS (Q62270)	<u>Kinases</u><br>B1 ABL1 (P00519)<br>B2 ABL2 (P42684)<br>B3 FER (PI6591)<br>B4 FES (P07332)<br>B5 TXK (P42681)<br>B6 ITK (Q08881)<br>B7 BTK (Q06187)<br>B8 TEC (P42680)<br>B9 BMX (P51813)<br>B10 CSK (P41240)	<u>Kinases</u><br>C1 SYK-C (Q5T6N8)<br>C2 SYK-N (Q5T6N8)<br>C3 ZAP70-C (P43404)<br>C4 ZAP70-N (P43404)<br>C5 MATK (P42679)<br><u>Phosphatases</u><br>C6 PTPN11-C (Q06124)<br>C7 PTPN11-N (Q06124)<br>C8 PTPN6-C (P29350)<br>C9 PTPN6-N (P29350)	<u>Scaffolds</u><br>D1 BLNK (Q8WV28)<br>D2 MIST (Q7Z7G1)<br>D3 SHC1 (P29353)<br>D4 SHC2 (P98077)<br>D5 SHC3 (Q92529)<br>D6 SHC4 (Q8IYW3<br>D7 SLNK (Q7Z4S9)<br>D8 SLP76 (Q13094)<br><u>Signal Regulation</u><br>D9 APS (O14492)<br>D10 SH2D2A (Q9NP31)	
<u>Signal Regulation</u><br>E1 DAPP1 (Q9UN19)<br>E2 HSH2D (Q96JZ2)<br>E3 GRB7 (Q14451)<br>E4 GRB10 (Q13322)<br>E5 GRB14 (Q14449)<br>E6 SHB (Q15464)<br>E7 SHD (Q96IW2)<br>E8 SHE (Q5VZ18)<br>E9 SHF (Q96IE8)<br>E10 SH2B(Q9NRF1)	<u>Adapters</u><br>F1 GRAP (Q13588)<br>F2 GADS/GRAP2 (O75791)<br>F3 GRB2 (P62993)<br>F4 CRK (P46108)<br>F5 CRKL (P41240)<br>F6 NCK1 (P16333)<br>F7 NCK2(O43639)<br>F8 SLAP (Q13239)<br>F9 SLAP2 (Q9H6Q3)<br><u>Chromatin Remodeling</u><br>F10 SUPT6H (Q7KZ85)	<u>Cytoskeletal Regulation</u><br>G1 BRDG1/STAP1 (Q9ULZ2)<br>G2 SH3BP2 (P78314)<br>G3 SH2D1A (O60880)<br>G4 SH2D1B (O14796)<br>G5 TNS1 (Q9HBL0)<br>G6 TNS2 (Q76MW6)<br>G7 TNS3 (Q8IZW7)<br>G8 TNS4 (Q8IZW8)<br><u>Phospholipid Signaling</u><br>G9 SHIP1 (O00145)<br>G10 SHIP2 (O15357)	<u>Phospholipid Signaling</u><br>H1 PIK3R1_C (P27986)<br>H2 PIK3R1_N (P27986)<br>H3 PIK3R2_C (O00459)<br>H4 PIK3R2_N (O00459)<br>H5 PIK3R3_C (Q92569)<br>H6 PIK3R3_N (Q92569)<br>H7 PLCg1-C (P19174)<br>H8 PLCg1-N (P19174)<br>H9 PLCg2-C (P16885)<br>H10 PLCg2-N (P16885)	<u>Small GTPase Signaling</u><br>I1 RASA1-N (P20936)<br>I2 RASA1-C (P20936)<br>I3 NSP1/SH2D3A(Q9BRG2)<br>I4 NSP1/BCAR3 (O75815)<br>I5 RIN1 (Q13671)<br>I6 RIN3 (Q8TB24)<br>I7 VAV1(P15498)<br>I8 VAV2 (P52735)<br>I9 VAV3 (Q9UKW4)<br>I10 CHN1 (P15882)

Plasmids were kindly provided by Dr. Shawn Li, Department of Biochemistry, University of Western Ontario
